# Involvement of gliadin, a component of wheat gluten, in increased intestinal permeability leading to non-steroidal anti-inflammatory drug-induced small-intestinal damage

**DOI:** 10.1371/journal.pone.0211436

**Published:** 2019-02-20

**Authors:** Sunao Shimada, Tetsuya Tanigawa, Toshio Watanabe, Akinobu Nakata, Naoki Sugimura, Shigehiro Itani, Akira Higashimori, Yuji Nadatani, Koji Otani, Koichi Taira, Shuhei Hosomi, Yasuaki Nagami, Fumio Tanaka, Noriko Kamata, Hirokazu Yamagami, Masatsugu Shiba, Yasuhiro Fujiwara

**Affiliations:** 1 Department of Gastroenterology, Osaka City University Graduate School of Medicine, Osaka, Japan; 2 SAMURAI GI Research Centre, Osaka City University Graduate School of Medicine, Osaka, Japan; Tulane University, UNITED STATES

## Abstract

Gliadin, a component of wheat gluten known to be an important factor in the etiology of celiac disease, is related to several other diseases through its enhancing effect on intestinal paracellular permeability. We investigated the significance of gliadin in non-steroidal anti-inflammatory drug (NSAID)-induced small-intestinal damage in mice. 7-week-old C57BL/6 male mice were divided into the following groups: standard diet group, in which mice were fed with wheat-containing standard rodent diet (CE-2); gluten-free diet group, in which mice were fed with gluten-free diet (AIN-76A); and gliadin-administered group, in which mice fed with gluten-free diet were administered with gliadin (~250 mg/kg BW). Each group was subdivided into negative, healthy control group and NSAID-treated group. To some mice fed with gluten-free diet and administered with gliadin, epidermal growth factor receptor (EGFR) tyrosine kinase inhibitor was administered for clarification of the significance of EGFR in NSAID-induced small intestinal damage and intestinal permeability. In mice fed with a gluten-free diet, indomethacin or diclofenac induced very mild mucosal damage in the small intestine compared with that in mice fed with a wheat-containing standard diet. Gliadin exacerbated the NSAID-induced small-intestinal damage in mice fed with a gluten-free diet. With the administration of indomethacin, MPO activity, a marker of neutrophil infiltration into the mucosa and mRNA expression level of tumor necrosis factor α and interleukin-1β in the small intestine were higher in the gliadin-administered mice. Gliadin increased the intestinal paracellular permeability without indomethacin administration (4.3-fold) and further increased the permeability after indomethacin administration (2.1-fold). Gliadin induced phosphorylation of epidermal growth factor receptor (EGFR) in small-intestinal tissues, and erlotinib (an EGFR tyrosine kinase inhibitor) attenuated the indomethacin-induced intestinal damage and permeability exacerbated by gliadin, accompanied by inhibition of EGFR phosphorylation. These results suggest that gliadin plays an important role in the induction and exacerbation of NSAID-induced small-intestinal damage, and that increase in intestinal permeability via the EGFR signalling pathway is involved in its mechanism.

## Introduction

The recent widespread use of video capsule endoscopy and balloon-assisted enteroscopy revealed that non-steroidal anti-inflammatory drugs (NSAIDs) induce small-intestinal damage.[1–4] Our recent study showed that 25% of patients with rheumatoid arthritis who took NSAIDs for more than 3 months had mild small-intestinal damage, and more important, 27.8% of patients had severe damage and decreased hemoglobin levels.[5] Although misoprostol and rebamipide are reported to be potential therapeutic and prophylactic agents for NSAID-induced small-intestinal damage,[6, 7] clarification of dietary factors associated with NSAID-induced small intestinal damage is also important for making therapeutic and prophylactic strategies against the disease as well as chemopreventive strategy.

In the pathophysiology of NSAID-induced small-intestinal damage, increase in mucosal permeability is one of the key mechanisms other than cyclooxygenase-mediated process.[8] In gastrointestinal tract, NSAIDs interact with phospholipids of cellular membrane and intracellular mitochondrial oxidative phosphorylation, which initiates biochemical changes that impair function of the mucosal barrier, which results in increase in intestinal permeability. Increased mucosal permeability induced by NSAIDs promotes recruitment of inflammation-inducing molecules such as lipopolysaccharide (LPS)[9] and bile[10, 11] from the small-intestinal lumen to the mucosa and submucosa, and proinflammatory molecules stimulate immune cells, triggering inflammatory reactions.[8] In clinical studies, it was also revealed that NSAID increases intestinal permeability in humans[12, 13]. Therefore, control of small-intestinal permeability could be the therapeutic and prophylactic target for NSAID-induced small-intestinal damage.

Wheat gluten is a complex of albumin, globulin, gliadin and glutenin.[14] Gliadin is a well-known pathogenic factor for celiac disease.[15] Other than celiac disease, recent studies suggested that gliadin may have a role in a variety of diseases,[16, 17] such as type 1 diabetes,[18] rheumatoid arthritis[19], primary Sjögren's syndrome[19], and multiple sclerosis[20]. Stimulation of production of proinflammatory cytokines such as interleukin (IL)-1β and tumor necrosis factor-α, IL-6,-8, -15, and monocyte chemotactic protein-1[21–23], and increase in intestinal permeability[17] are involved in the mechanism by which gliadin induce a variety of diseases. It was reported that in *in vitro* experiment gliadin induce the release of zonulin [24], and another study showed that zonulin transactivate epidermal growth factor receptor (EGFR) and its activation increase intestinal permeability via modulation of tight junction proteins.[25] Collectively, we hypothesized that gliadin could induce small intestinal permeability via EGFR activation.

As increased permeability is also associated with the pathophysiology of NSAID-induced small-intestinal damage,[26, 27] we examined the significance of gliadin in NSAID-induced small-intestinal damage.

## Materials and methods

### Animals

Seven-week-old specific pathogen-free (SPF) male C57BL/6 mice were purchased from Charles River Japan (Yokohama, Japan). The animals were kept in the filtered-air ventilated cage rack and were fed with a wheat-containing standard rodent diet (CE-2; CLEA Japan Inc., Tokyo, Japan) until the experiment. All mice were allowed free access to diet and water, with no special indications. When performing invasive procedures including cardiac puncture and euthanasia, anesthesia was always conducted using isoflurane anesthetizer (MK-A110D, Muromachi Kikai, Tokyo, Japan). Anesthesia was induced at 4% isoflurane with 20% oxygen using chamber and maintained at 1.5–2.75% isoflurane with 20% oxygen using anesthetic mask. Euthanasia was performed by cervical dislocation with deep euthanasia. This study was carried out in strict accordance with the recommendations in the Guide for the Care and Use of Laboratory Animals of the National Institutes of Health. All experiments were carried out with confirmation of The Regulations on Animal Experiments and with approval of the Institutional Animal Care and Use Committee of Osaka City University Graduate School of Medicine. All invasive procedures were performed under isoflurane anesthesia, and all efforts were made to minimize suffering.

### Standard diet (SD) and gluten-free diet (GFD)

After arrival, all mice were fed a standard rodent diet (CE-2), which contains wheat for carbohydrate source, for one-week acclimatization. Mice of the SD group continued the CE-2 diet, and mice of the GFD group were converted to an AIN-76A rodent diet (gliadin-free standardized diet, purchased from CLEA Japan), continued for 5 days for washout of enteric luminal gliadin.

### Reagents

Gliadin from wheat was purchased from Tokyo Chemical Industry (Tokyo, Japan). Indomethacin, diclofenac, zein, and Evans blue were purchased from Wako Pure Chemical Inc. (Osaka, Japan). Fluorescein isothiocyanate (FITC)-dextran (average molecular weight 3000–5000) (#FD4) and monoclonal anti-β-actin antibody (#AC-15) were purchased from Sigma-Aldrich (St. Louis, MO, USA). Erlotinib, a tyrosine kinase inhibitor that acts on EGFR, was purchased from Cayman Chemical (Ann Arbor, MI, USA). Anti-EGFR antibody and anti-phosphorylated-EGFR (Tyr1068) (p-EGFR) antibody were purchased from Cell Signaling Technology (Beverly, MA, USA).

### Analysis of gliadin fractionation and determination of the amount of gliadin in the diet

Gliadin is classified into four subgroups as α-, β-, γ- and ω-gliadin.[28] And each subgroup has been mentioned to have a different biochemical activity. Especially α-gliadin is reported to have cytotoxicity[29] and synthetic α-gliadin peptide is often used at *in vitro* studies[24, 30, 31]. Before starting experiments, we determined the profile of gliadin we planned to use.

Gliadin was dissolved in 0.01 mol/L HCl and adjusted to 2 mg/mL and pH 6.8 with saline and NaOH. The solution was boiled for 5 min with Sample Buffer Solution (Wako Pure Chemical). A 20 μg of protein was loaded onto 15% sodium dodecyl sulfate (SDS)-polyacrylamide gel. DynaMarker Protein MultiColor Stable (BioDynamics Laboratory, Tokyo, Japan) was used to monitor protein migration and molecular weights during electrophoresis. Gliadin and protein marker were separated using electrophoresis (for 60 min at 180 V, room temperature) with running buffer (25 mmol/L Tris, 192 mmol/L glycine and 0.1% SDS), and the gel was stained with Coomassie brilliant blue according to the manufacturer’s instructions (QuickBlue Staining Solution, BioDynamics Laboratory, Tokyo, Japan).

Concentration of gliadin in CE-2 diet and AIN-76A diet was determined by ELISA assay according to manufacturer’s instruction (Wheat/Gluten (Gliadin) ELISA Kit II, Morinaga, Institute of Biological Science, Inc., Yokohama, Japan).

### Induction and assessment of small-intestinal damage induced by indomethacin and diclofenac

The animal experiments were conducted in accordance with our previous report.[32, 33] 10 mg/kg body weight (BW) of indomethacin with vehicle (0.5% carboxymethylcellulose [CMC]) was orally administered to nonfasted animals by gavage. Mice were sacrificed 24 h later. For evaluation of macroscopic damage, 1% Evans blue was injected intravenously 1 h before sacrifice in order to delineate the mucosal damage, and the small intestine was collected and opened along the anti-mesenteric attachment side of the lumen. In some experiments, 60 mg/kg BW diclofenac with vehicle (0.5% CMC) was orally administered instead of indomethacin. To examine the effect of gliadin on the intestinal damage induced by indomethacin, vehicle (0.01 mol/L HCl) or gliadin was administered orally in divided doses (0–250 mg/kg BW per time) at 24, 12, and 0 h before NSAID administration.

To examine whether the effect of gliadin at a low dose on NSAID-induced small intestinal damage is dependent on the dose of indomethacin, some mice fed with GFD and administered orally with low-dose gliadin (12.5 mg/kg BW per time) were given 10, 20 and 30 mg/kg BW of indomethacin and the lesion index was evaluated.

The schema of the animal experiment protocol is shown in S2 and S4 Figs.

The areas (mm^2^) of macroscopically visible lesions were measured, summed per small intestine, and expressed as the lesion index. For histological evaluation, each of the small-intestinal tissue samples that showed typical mucosal damage was fixed with 10% buffered formalin, and 4-μm-thick tissue sections mounted on a glass slide were stained with hematoxylin and eosin. Two investigators (TT and Yuji N) scored at least 10 random villi at injured areas under a white-light microscope, in a blinded fashion. We adopted the histological scoring system previously described.[7]

### Measurement of myeloperoxidase (MPO) activity

MPO activity, a marker of neutrophil infiltration into the mucosa, was assayed according to a previously described method.[9] In brief, small-intestinal tissues from the ileum were homogenized on ice in 50 mM potassium phosphate buffer (pH 6.0) containing 0.5% hexadecyltrimethylammonium bromide. Each suspension was frozen once in dry ice, dissolved using an ultrasound generator, and centrifuged. Then, the MPO activity in the supernatant was assayed with a spectrophotometer. One unit of MPO activity was defined as that degrading 1 μmol of peroxide per minute at 25°C. Results were expressed as units per gram tissue.

### Real-time quantitative reverse transcription polymerase chain reaction (RT-PCR) analyses

Total RNA was isolated from small-intestinal tissue (1.5-cm length of the ileum) using ISOGEN-2 kit (Nippon-gene, Tokyo, Japan). Complementary DNA was acquired using High-Capacity RNA-to-cDNA Kit (ThermoFisher Scientific Inc., Waltham, MA, USA). RT-PCR was performed using Applied Biosystems 7500 Fast RT-PCR system with ABI TaqMan Fast Universal PCR Master Mix (ThermoFisher Scientific Inc.). The thermal cycling method was 50 cycles of amplification at 95°C for 15 s and 60°C for 1 min. The mRNA expression levels for interleukin-1β (IL-1β; encoded by *Il1b*) and tumor necrosis factor α (TNFα; encoded by *Tnfα*) were quantified using real-time RT-PCR and standardized to TaqMan glyceraldehyde-3-phosphate dehydrogenase (ThermoFisher Scientific Inc.). The mRNA levels were expressed as ratios of the mean value for vehicle-treated mice. The sequences of PCR primers and TaqMan probes are as follows: for the mouse *Il1b*, the sense primer was 5′-GCTGCTACTCATTCACTGGCAA-3′, the anti-sense primer was 5′-TGCTGCTGGTGATTCTCTTGTA-3′, and the TaqMan probe was 5′-FAM-ATGATCCCAATGAGTCGGCTGGAGA-TAMRA-3′; for the mouse *Tnfα*, the sense primer was 5′-TCATGCACCACCATCAAGGA-3′, the anti-sense primer was 5′-GAGGCAACCTGACCACTCTCC-3′, and the TaqMan probe was 5′-FAM-AATGGGCTTTCCGAATTCACTGGAGC-TAMRA-3′.

### Measurement of intestinal permeability by using FITC-dextran

Intestinal permeability was determined using a previously reported protocol.[34] FITC-dextran (44 mg per 0.44 mL per 100 g BW) dissolved in phosphate-buffered saline (PBS) was orally gavaged to the mice fasted for 12 h. After 4 h, blood was collected through cardiac puncture before euthanasia. Blood was kept on ice for 1 day, and centrifuged (4°C, 15,000 rpm, 10 min) to collect the serum. The concentration of FITC in the serum was determined using a spectrophotofluorometer with an excitation of 485 nm and an emission of 528 nm, with a standard curve made with progressively diluted FITC-dextran. The result was corrected by subtracting that of the blank sample (serum from the vehicle [PBS]-administered mice). All procedures were carried out in the darkroom.

We measured the serum FITC-dextran concentrations among the mice with or without gliadin administered by gavage, and mice with or without indomethacin administered by gavage. We administered gliadin (250mg/kg BW per time, 25 mg/mL dissolved in 0.01 mol/L HCl) or vehicle (0.01 mol/L HCl) orally at 24, 12, and 0 h before oral indomethacin (10 mg/kg BW, 1mg/mL dissolved in 0.5% CMC) or vehicle (0.5% CMC) administration. And 4 h after oral indomethacin or vehicle administration, FITC-dextran was orally gavaged. To confirm whether there is a difference in absorption efficiency based on the protein density of the gut lumen, we used zein, a major storage protein of corn, as a negative control protein for gliadin [31] The schema of the animal experiment protocol is shown in S3 and S4 Figs.

### Effects of EGFR phosphorylation on intestinal damage

Vehicle (PBS) or erlotinib (50 μg/g BW) was administered intraperitoneally 24 h before indomethacin administration. The schema of the animal experiment protocol is shown in S4 Fig. Small-intestinal tissues (1.5-cm length of the ileum) were rinsed in normal saline and stored at -80°C until analysis.

### Western blotting analysis

The samples were homogenized in 500 μL of NP-40 lysis buffer, containing 25 mM 4-(2-hydroxyethyl)piperazine-1-ethane-sulfonic acid, 150 mM NaCl, 4 mM ethylenediaminetetraacetic acid, 1% NP-40, cOmplete Mini (1 tablet per 10 mL; Thermo Fisher Scientific Inc.), and PhosSTOP (1 tablet per 10 mL, adjusted to pH 7.4 with NaOH; Roche Applied Science, Indianapolis, IN, USA), on ice. The homogenized samples were gently rotated for 30 min at 4°C and centrifuged at 10,000*g* for 30 min at 4°C. The supernatant was collected and transferred to other microcentrifuge tubes. Protein concentrations were measured with Pierce BCA Protein Assay Kit (Thermo Fisher Scientific Inc.). Samples were boiled for 5 min with Sample Buffer Solution (Wako Pure Chemical) and loaded onto the 15% or 7.5% SDS-polyacrylamide gels. DynaMarker Protein MultiColor Stable was also loaded to monitor protein migration and molecular weights during electrophoresis. A 20 μg of protein and protein marker were separated using electrophoresis (for 60 min at 180 V, room temperature) with running buffer (25 mmol/L Tris, 192 mmol/L glycine and 0.1% SDS) and transferred to an Immune-Blot poly-vinylidene difluoride membrane (Bio-Rad). Each membrane was blocked with blocking buffer (5% skim milk in Tris-buffered saline [TBS]-T; TBS containing 0.1% Tween-20) for 1 h at room temperature and rinsed 3 times with TBS-T. Membranes were incubated with primary antibodies (anti-EGFR and anti-p-EGFR at 1:1000 dilution, and β-actin at 1:5000 dilution) in blocking buffer overnight at 4°C. After rinsing 3 times with TBS-T, membranes were incubated with the horseradish peroxidase-conjugated secondary antibodies (#NA934-1ML, GE Healthcare, Little Chalfont, UK) at 1:1000 dilution in blocking buffer for 1 h at room temperature. After rinsing 3 times with TBS-T, membranes were exposed to ECL Prime Western Blotting Detection Reagent (GE Healthcare). Chemiluminescence was detected with ImageQuant LAS 4000 Mini (GE Healthcare) and quantified using the software ImageQuant TL Analysis Toolbox (GE Healthcare) and ImageJ (Rasband, W.S., ImageJ; US National Institutes of Health, Bethesda, MD, USA). The expression levels of EGFR and phosphorylated-EGFR were normalized to that of β-actin. All data were expressed as the percentage change from control. Independent experiments were performed at least twice.

### Statistical analysis

Values were expressed as mean ± standard error of mean. One-way analyses of variance were used to test for significance of differences among treatment group means, and results were analyzed using Fisher’s protected least significant difference test. Differences with *p*-values <0.05 were considered statistically significant.

## Results

### Gliadin fractionation with Coomassie brilliant blue staining of SDS-polyacrylamide gels and concentration of gliadin in the diet

Fractionation of the gliadin used in the present study yielded staining bands with molecular weights mainly between 30 and 42 kDa, which correspond to α/β- and γ-gliadins.[35] High molecular weight fractions, which represent ω-gliadin, were observed less than low molecular weight fractions (S1 Fig).

The amount of gliadin in CE-2 diet was 640.3 ppm, whereas gliadin was not detected in AIN-76A diet.

### Gliadin exacerbates NSAID-induced small-intestinal damage and inflammation

**Fig 1:**
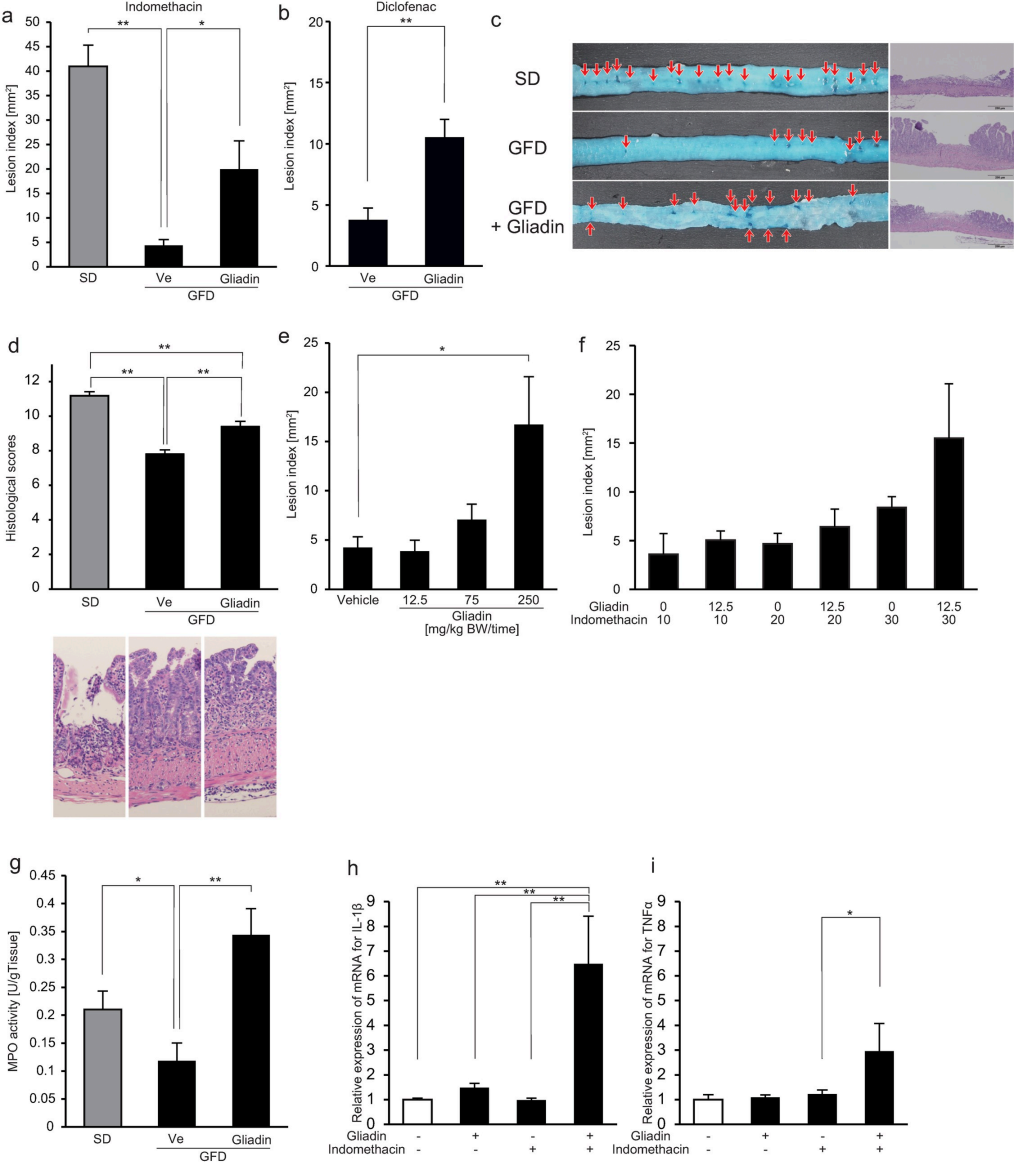
Effect of gliadin on small-intestinal damage and inflammation induced by non-steroidal anti-inflammatory drugs (NSAIDs). (a,b) Lesion indices in the small intestine after NSAID administration (a: indomethacin, b: diclofenac) in mice fed with a standard diet (SD) or a gluten-free diet (GFD). In mice fed with the GFD, we examined the effect of gliadin administration on the lesion indices. Lesion index was defined as the total area of deep-dyeing concavities of the mucosa delineated by intravenous injection of Evans blue dye. n = 4–6. (c) Typical macroscopic and microscopic images of indomethacin-induced small-intestinal damage and the effect of gliadin on the damage. The lesion is macroscopically visualized as areas of the deep-dyeing concavities of the mucosa delineated by intravenous injection of Evans blue dye (arrows). Bars in histological images: 200 μm. (d) Histological scores of the small intestine after indomethacin administration. Histological scores were estimated on a scale from 0 to 13 according to microscopic findings. n = 5. The photo images are typical high-power field images of mucosal break and infiltration of inflammatory cells into the mucosa. (e) Dose-dependent effect of gliadin on lesion indices after indomethacin administration. n = 7. (f) Lesion indices in the small intestine after 10, 20 or 30 mg/kg BW indomethacin administration in mice fed with GFD and administered orally with low dose gliadin (0 (vehicle) or 12.5 mg/kg BW per time). Lesion index was defined as the total area of deep-dyeing concavities of the mucosa delineated by intravenous injection of Evans blue dye. n = 5–6. (g) Myeloperoxidase (MPO) activities of small-intestinal tissue after indomethacin administration. n = 8. (h, i) mRNA expression levels of interleukin-1β (IL-1β) and tumor necrosis factor α (TNFα) in small-intestinal tissues as determined using quantitative reverse transcription polymerase chain reaction. The mRNA expression levels were shown as ratios relative to the mean value for small-intestinal tissues from untreated mice in each group. n = 8. All data were expressed as mean ± standard error of mean. * *p* < 0.05, ** *p* < 0.01.

Macroscopic damages induced by indomethacin administration were recognized as deep-dyeing concavities of the mucosa. Compared with the SD group, in which mice were fed the SD we usually use in experimental studies on NSAID-induced small-intestinal damage, the lesion indices were markedly reduced in the GFD group (Fig 1A and 1C). In the GFD group, gliadin increased the lesion indices compared with those in vehicle-treated mice (Fig 1A and 1C). Similar to the indomethacin-administered GFD group, lesion indices were increased by gliadin administration in the diclofenac-administered GFD group (Fig 1B). The histological scores of the SD and GFD groups administered with indomethacin showed a similar tendency of the lesion indices (Fig 1D). In the GFD group, administration of gliadin increased the lesion indices in the GFD group in a dose-dependent manner (Fig 1E).

In mice administered with gliadin at a low dose (12.5 mg/kg), the lesion index tended to be higher in mice with high dose of indomethacin (30 mg/kg) than mice with 10 and 20 mg/kg of indomethacin, but it did not reach statistical significance (Fig 1F). These results indicate that the dose of gliadin is important for induction of NSAID-induced small intestinal damage.

Gliadin increased the MPO activities of small-intestinal tissue in the GFD group administered with indomethacin (Fig 1G). The mRNA expression level of IL-1β in the small intestine of the GFD group increased with a combination of gliadin and indomethacin administrations (Fig 1H). With gliadin administration alone, the mRNA expression level of IL-1β increased but without a statistically significant difference (Fig 1H). Concerning TNFα, gliadin increased the mRNA level only with indomethacin administration (Fig 1I).

### Gliadin increases intestinal paracellular permeability and further increases the permeability after indomethacin administration

**Fig 2:**
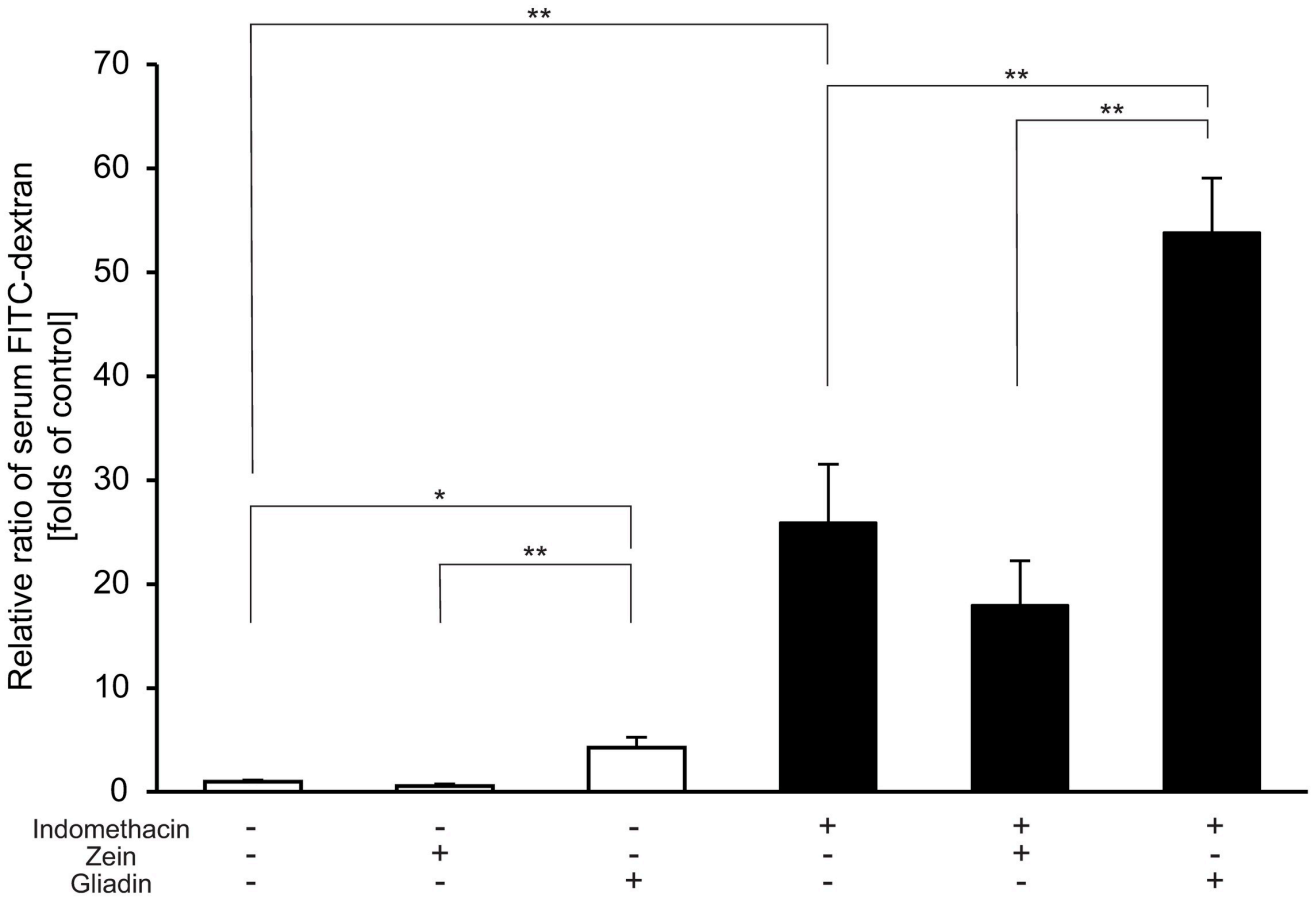
Effect of gliadin on small-intestinal permeability without and with indomethacin administration determined according to relative ratios of the concentration of serum fluorescein isothiocyanate (FITC)-dextran. The fluorescence was measured using a spectrophotofluorometer, and the concentrations were determined through comparison with a standard curve of serially diluted FITC-dextran. The concentrations of serum FITC-dextran were expressed as ratios relative to the mean value from vehicle-administered mice. n = 5–8. All data were expressed as mean ± standard error of mean. * *p* < 0.05, ** *p* < 0.01.

Gliadin administration resulted in a 4.3-fold increase in intestinal paracellular permeability without the administration of indomethacin. Gliadin also increased the intestinal permeability in indomethacin-administered mice compared with that in vehicle-treated mice (2.1-fold). Zein did not affect the intestinal paracellular permeability with and without the administration of indomethacin (Fig 2).

### Exacerbating effect of gliadin was associated with EGFR phosphorylation

**Fig 3:**
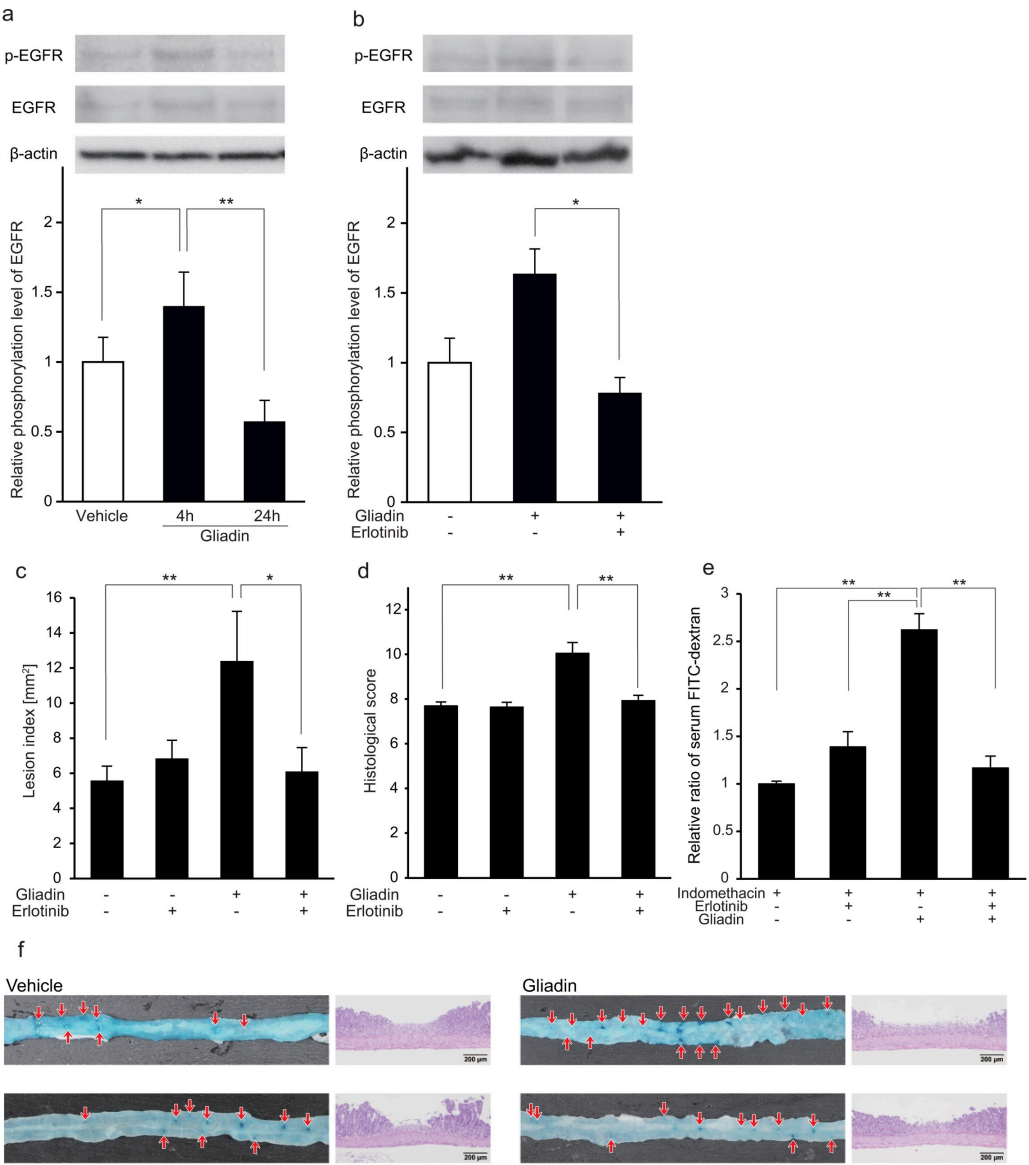
Role of phosphorylation of epidermal growth factor receptor (EGFR) on the exacerbating effect of gliadin on indomethacin-induced small-intestinal damage. (a) Time course of phosphorylation of EGFR assessed with Western blotting. The phosphorylation levels of EGFR were normalized to those of EGFR, and relative expression levels were expressed as ratios relative to the mean value for small-intestinal tissues from vehicle-treated mice in each experimental group. n = 5–6. (b) Effect of gliadin administration on phosphorylation of EGFR of small-intestinal tissue assessed with Western blotting. Small-intestinal tissues were obtained after 4 h of gliadin administration. The phosphorylation levels of EGFR were normalized to those of EGFR, and relative expression levels were expressed as ratios relative to the mean value for small-intestinal tissues from vehicle-treated mice in each experimental group. n = 5–6. (c) Lesion indices after indomethacin administration with and without intraperitoneal administration of erlotinib. n = 4–6. (d) Histological scores of the small intestine after 24 h of indomethacin administration. Histological scores were estimated on a scale from 0 to 13 according to microscopic findings. n = 5. The photo images are typical high-power filed image of mucosal break and infiltration of inflammatory cells into the mucosa. (e) Effect of erlotinib administration on indomethacin. The concentrations of serum FITC-dextran were expressed as ratios relative to the mean value from indomethacin-and-vehicle-administered mice in each experiment group. n = 5–8. (f) Macroscopic and microscopic photos of small-intestinal lesions after 24 h of indomethacin administration. Lesions were recognized macroscopically as deep-dying blue spots in the small intestine (red arrows). Bars in histological images: 200 μm. All data were expressed as mean ± standard error of mean, *p < 0.05, **p < 0.01.

We clarified the involvement of EGFR phosphorylation in the mechanism of exacerbation of indomethacin-induced small-intestinal damage by gliadin. In western blot time-course analysis, the relative phosphorylation rate of EGFR in small-intestinal tissue after a single administration of gliadin increased 4 h later and disappeared after 24 h (Fig 3A). The relative phosphorylation rate of EGFR was increased by gliadin administration, and it was suppressed by the administration of erlotinib (Fig 3B).

Based on the above results, we investigated whether erlotinib influences the enhancing effect of gliadin on indomethacin-induced small-intestinal damage. The lesion indices with indomethacin administration without erlotinib increased in gliadin-administered mice, and erlotinib attenuated the small-intestinal lesion indices with statistical significance (Fig 3C and 3F). The histological scores showed a similar tendency to that of the lesion indices (Fig 3D). In FITC-dextran experiments with indomethacin administration, erlotinib attenuated paracellular permeability in the gliadin-administered group (Fig 3E). Moreover, there was no significant difference in permeability between the vehicle-administered groups with or without erlotinib (Fig 3E).

## Discussion

In the present study, we demonstrated that gliadin exacerbates NSAID-induced small-intestinal damage. Gliadin increased intestinal paracellular permeability through the upregulation of EGFR phosphorylation, which is involved in the mechanism by which gliadin exacerbates NSAID-induced small intestinal damage.

Assessment of dietary factors associated with NSAID-induced small-intestinal damage is important in understanding its etiology and in developing therapeutic and prophylactic strategies against the disease; however, these dietary factors remain unknown. In the present study, we demonstrated that GFD induces minimal NSAID-induced small-intestinal damage compared with a standard diet, which contains wheat gluten, and that gliadin induces small intestinal damage in mice receiving GFD. This indicates that NSAID-induced small-intestinal damage could be prophylactically suppressed by avoiding the intake of gluten.

NSAIDs increase the small-intestinal mucosal permeability, and a variety of intraluminal pathogens, such as enterobacteria-derived endotoxins, LPS, and other toxic agents, pass through the epithelial barrier and stimulate mucosal and submucosal inflammatory cells, initiating a cascade of inflammatory reactions and resulting in mucosal damage.[36] The molecules that increase small-intestinal permeability could exacerbate NSAID-induced small-intestinal damage. In the literature, bile acids,[37, 38] enterobacteria and its products such as LPS,[39, 40] and alarmins[41] were previously reported to increase small-intestinal permeability.

In recent years, it was revealed that gliadin induces a morbid increase in intestinal paracellular permeability in some pathological conditions other than celiac disease.[16, 17] In these pathophysiological conditions, the disease is believed to be caused by several intraluminal pathogens passing through the epithelial barrier that was loosened by gliadin.[17] In our experiments, gliadin administration without NSAIDs increased intestinal permeability compared with vehicle treatment; however, it did not increase the mRNA expression levels of IL-1β and TNF-α and did not induce visible small-intestinal damage. These results suggest that intestinal inflammation that is sufficient for the development of small-intestinal damage is not induced by gliadin itself; however, once a damaging agent such as an NSAID is administered, gliadin has the potential to exacerbate the mucosal damage.

In the present study, we showed that gliadin induces phosphorylation of EGFR in the small intestine accompanied by an increase in intestinal permeability. Inhibition of EGFR phosphorylation with a specific inhibitor abrogates the effect of gliadin on NSAID induced intestinal permeability. These results support a key role of EGFR in the increase in intestinal permeability by gliadin in the presence of NSAID. In some pathophysiological situations, EGFR phosphorylation is important for modulating increased permeability. Raimondi et al. reported that bile acid increased intestinal permeability through EGFR phosphorylation, in an *in vitro* model.[42] Transactivation of EGFR by an oxidant results in hyperpermeability in human colonic epithelial cells.[43] Therefore, it may be possible that phosphorylation of EGFR is the main effector of the gliadin-induced increase in intestinal permeability and exacerbation of NSAID-induced small-intestinal damage. Myosin light-chain kinase, which is mediated by extracellular signal-regulated kinase 1/2, a downstream signalling pathway of EGFR, is reported to be an effector in the downstream EGFR signalling responsible for intestinal hyperpermeability.[44]

The upstream mechanism by which gliadin exacerbates indomethacin-induced small-intestinal damage through the induction of EGFR phosphorylation remains unclear. One of the possible mechanisms is the involvement of zonulin[45]. Fasano et al. demonstrated that binding of gliadin with the chemokine receptor CXCR3 at the luminal surface of epithelial cells triggers a MyD88-dependent luminal zonulin release. It was also reported that zonulin transactivates epithelial EGFR through proteinase-activating receptor 2 activation, and this enhances the signalling to modulate tight junction proteins to disassemble intestinal tight junctions, followed by an increase in paracellular permeability.[24, 25, 42, 46, 47] Considering from these results, these mechanisms may be involved in the pathophysiology of deleterious effect of gliadin on NSAID-induced small intestinal damage. Oxidative stress may also be one of the other possible mechanisms. Previous reports showed that a particular gliadin peptide that accumulated in lysosomes induced oxidative stress in T84 and Caco-2 cell lines,[48] and that increased permeability in response to an oxidant was related to EGFR phosphorylation.[43] Further studies are needed to elucidate the specific gliadin-triggered signalling cascades that lead to increased epithelial permeability through the phosphorylation of EGFR in NSAID-induced small-intestinal damage.

Other pathophysiological situations in which increase in intestinal permeability is an important mechanism, such as stress-induced intestinal disorder,[49] inflammatory bowel disease,[49, 50] may be associated with the effect of gliadin on intestinal permeability. The clarification whether the result of our present study is extrapolated to other types of small intestinal diseases warrants further investigation.

In conclusion, our present study revealed that gliadin exacerbates NSAID-induced small-intestinal damage. Increase in small-intestinal permeability through phosphorylation of EGFR may be responsible for the mechanism by which gliadin exacerbates NSAID-induced small-intestinal damage. Selection of the GFD may be a good therapeutic and preventive strategy against NSAID-induced small-intestinal damage.

## Supporting information

S1 Checklist The ARRIVE Guidelines checklist. (DOCX)

S1 Fig Gliadin fractionation. (TIF)

S2 Fig Schema of the animal experiment protocols in this study. CMC: carboxymethylcellulose, G: oral administration of gliadin.
(TIF)

S3 Fig Schema of the animal experiment protocols in this study. CMC: carboxymethylcellulose, Z: oral administration of zein, G: oral administration of gliadin.
(TIF)

S4 Fig Schema of the animal experiment protocols in this study. G: oral administration of gliadin, Z: oral administration of zein.
(TIF)
