# Supplementary material for: Involvement of gliadin, a component of wheat gluten, in increased intestinal permeability leading to non-steroidal anti-inflammatory drug-induced small-intestinal damage
Source: PLoS One. 2019 Feb 20;14(2):e0211436. doi: 10.1371/journal.pone.0211436 (PMC6382145; doi:10.1371/journal.pone.0211436)

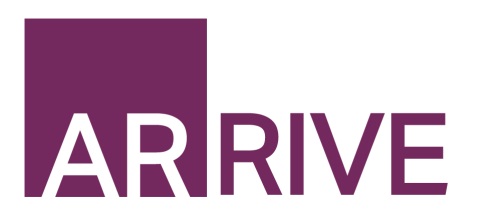


The ARRIVE Guidelines Checklist

Animal Research: Reporting In Vivo Experiments

Carol Kilkenny^1^, William J Browne^2^, Innes C Cuthill^3^, Michael Emerson^4^ and Douglas G Altman^5^

*^1^The National Centre for the Replacement, Refinement and Reduction of Animals in Research, London, UK, ^2^School of Veterinary Science, University of Bristol, Bristol, UK, ^3^School of Biological Sciences, University of Bristol, Bristol, UK, ^4^National Heart and Lung Institute, Imperial College London, UK, ^5^Centre for Statistics in Medicine, University of Oxford, Oxford, UK.*

|  | | ITEM | RECOMMENDATION | Section/ Paragraph |
| --- | --- | --- | --- | --- |
| 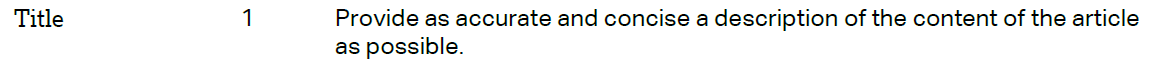 | | | Title. |  |
| 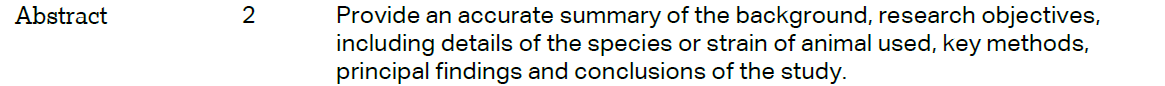 | | | Abstract. |  |
| INTRODUCTION | | |  |  |
| 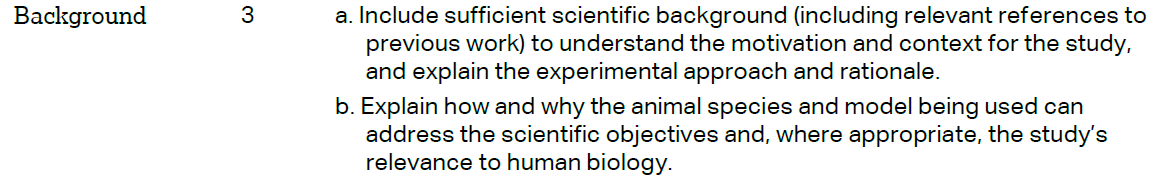 | | | Introduction. |  |
| 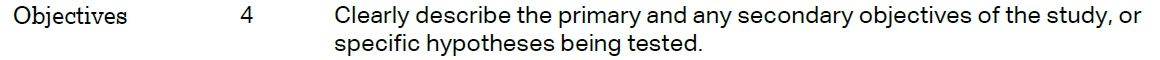 | | | Introduction. |  |
| METHODS | | |  |  |
| 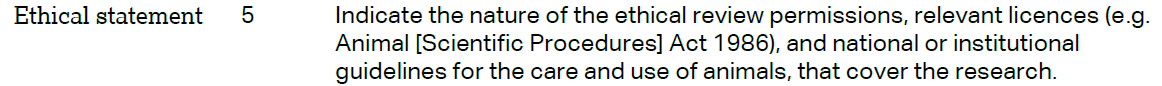 | | | Material and methods – Animals. |  |
| 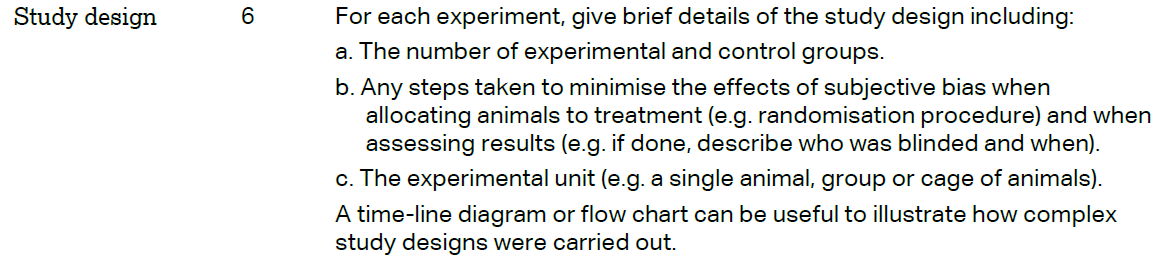 | | | Materials and methods – Animals.  Figure 1, 2 and 3.  Supporting file. |  |
| 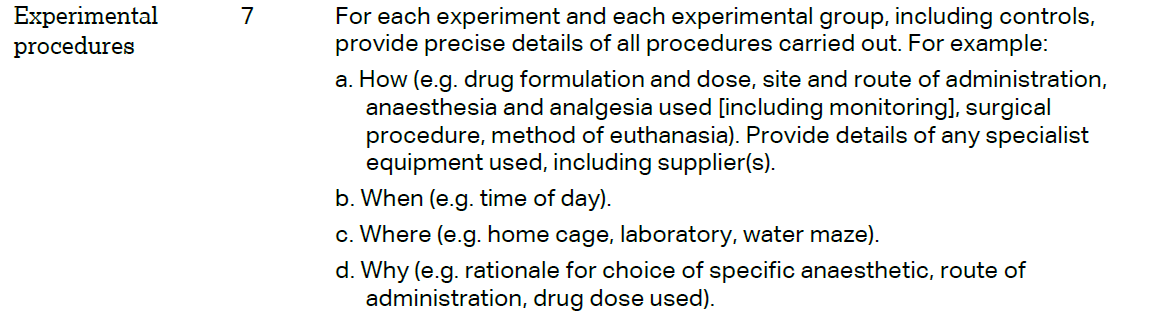 | | | Materials and methods. |  |
| 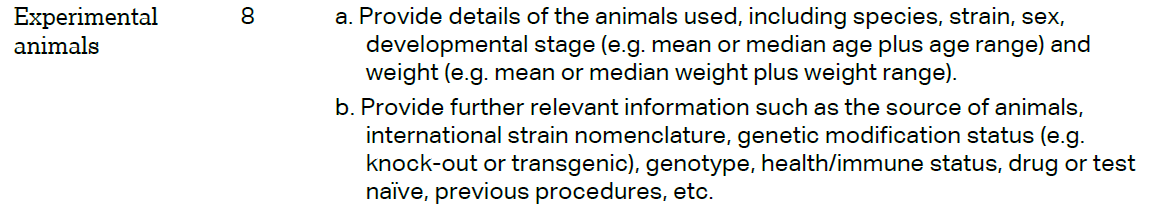 | | | Materials and methods – Animals. |  |

The ARRIVE guidelines. Originally published in *PLoS Biology*, June 2010^1^

| 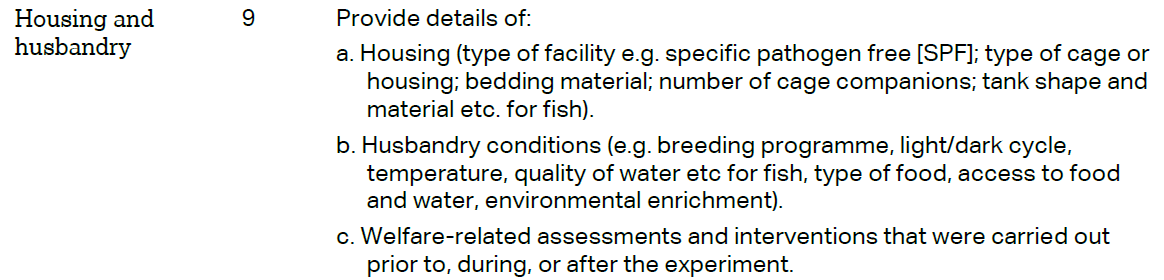 | Materials and methods. | |
| --- | --- | --- |
| 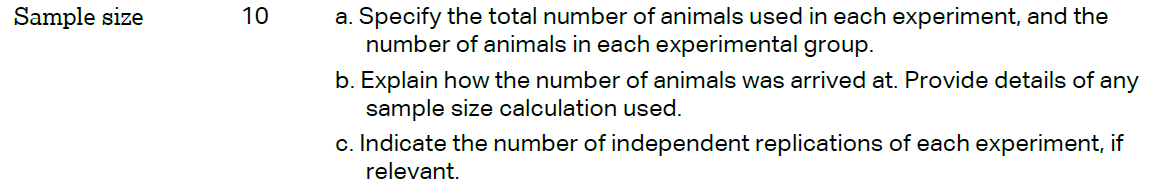 | Materials and methods – Animals.  Figure 1, 2 and 3. | |
| 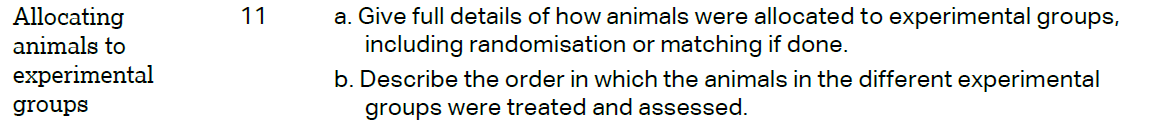 | Materials and methods – Animals. | |
| 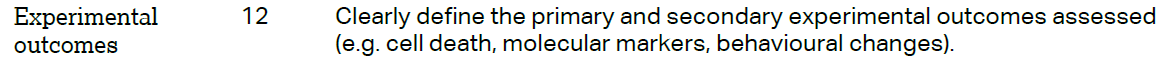 | Materials and methods. | |
| 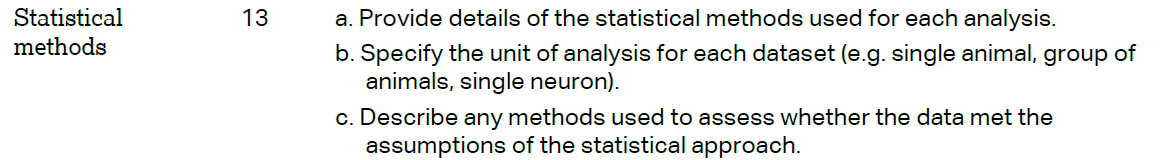 | Materials and methods – Statistical analysis. | |
| RESULTS |  | |
| 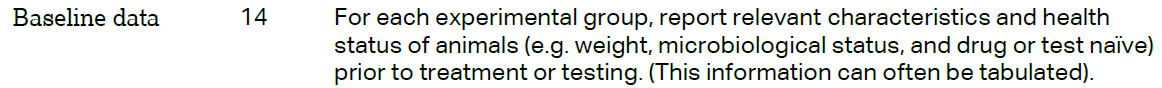 | Materials and methods – Animals. | |
| 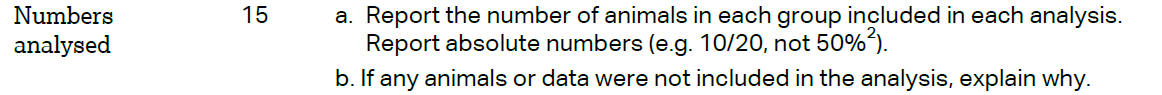 | Figure 1, 2 and 3. | |
| 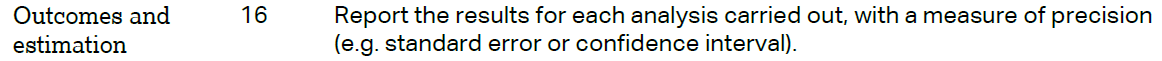 | Results.  Figure 1, 2 and 3. | |
| 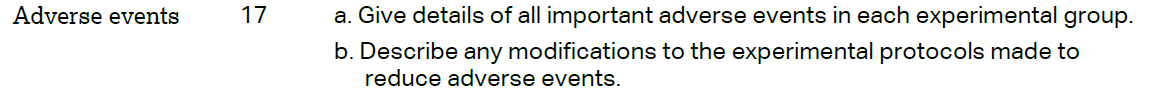 | N/A | |
| DISCUSSION |  | |
| 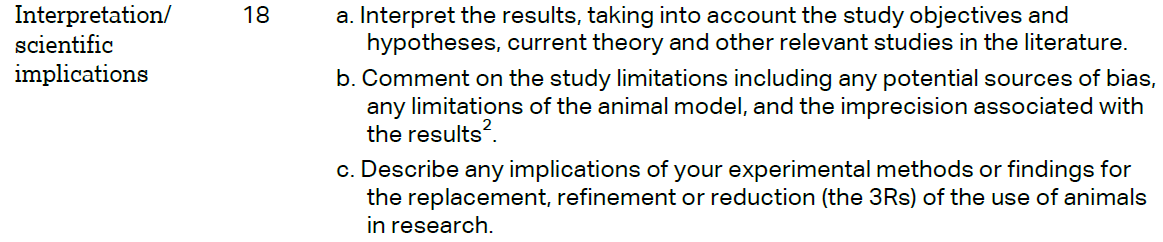 | Discussion. | |
| 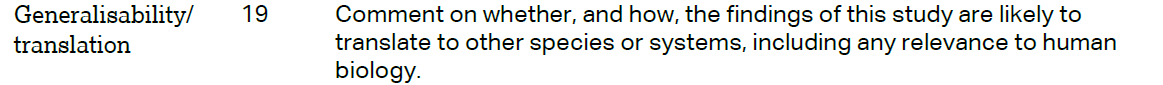 | Discussion. | |
| 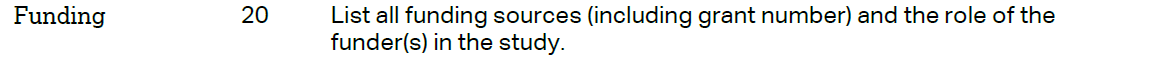 | | Funding. |


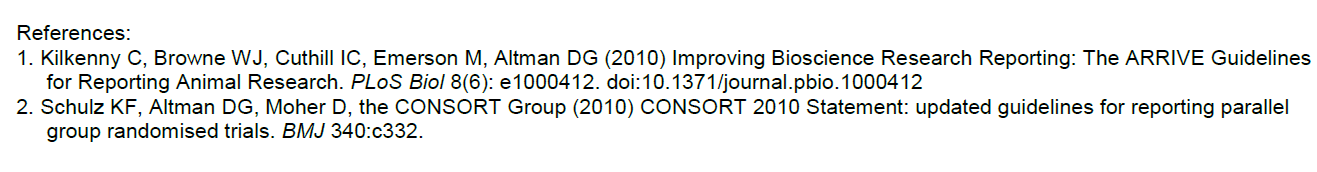

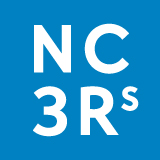

Supplement: S1 Checklist — (DOCX) [file pone.0211436.s001.docx]
