# Supplementary figures and images for: Involvement of gliadin, a component of wheat gluten, in increased intestinal permeability leading to non-steroidal anti-inflammatory drug-induced small-intestinal damage
Source: PLoS One. 2019 Feb 20;14(2):e0211436. doi: 10.1371/journal.pone.0211436 (PMC6382145; doi:10.1371/journal.pone.0211436)

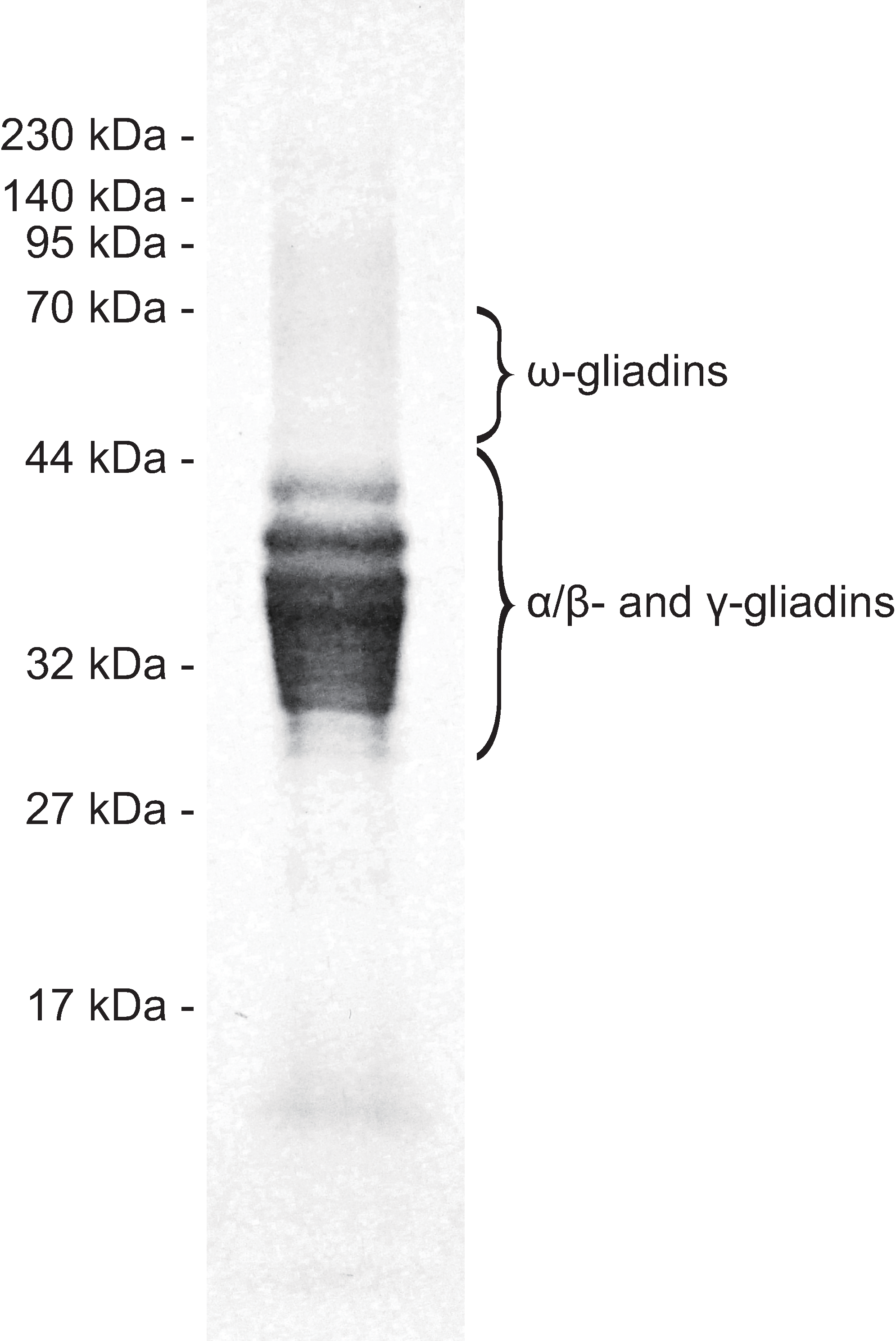

Supplement: S1 Fig — (TIF) [file pone.0211436.s002.tif]

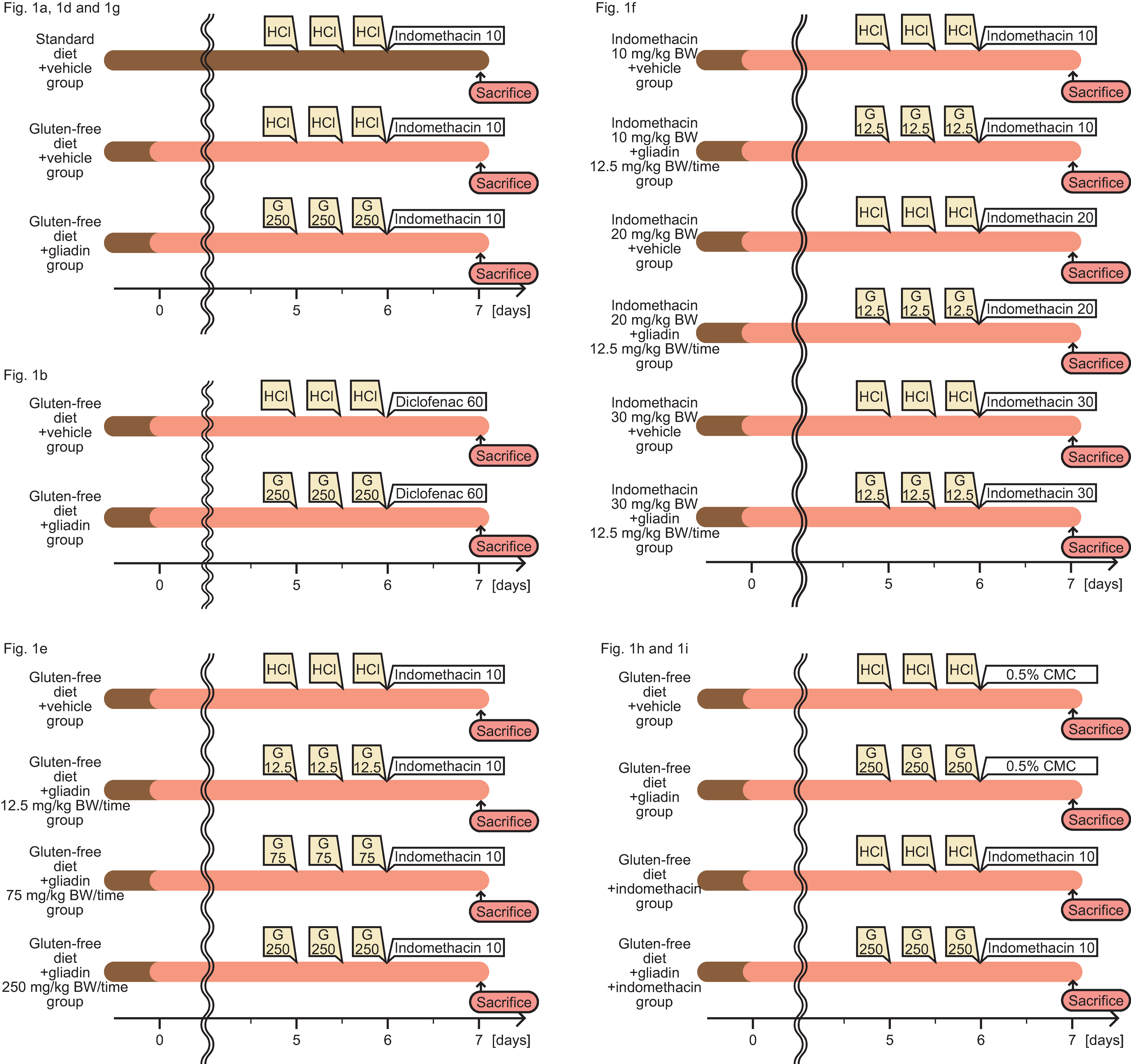

Supplement: S2 Fig — CMC: carboxymethylcellulose, G: oral administration of gliadin. (TIF) [file pone.0211436.s003.tif]

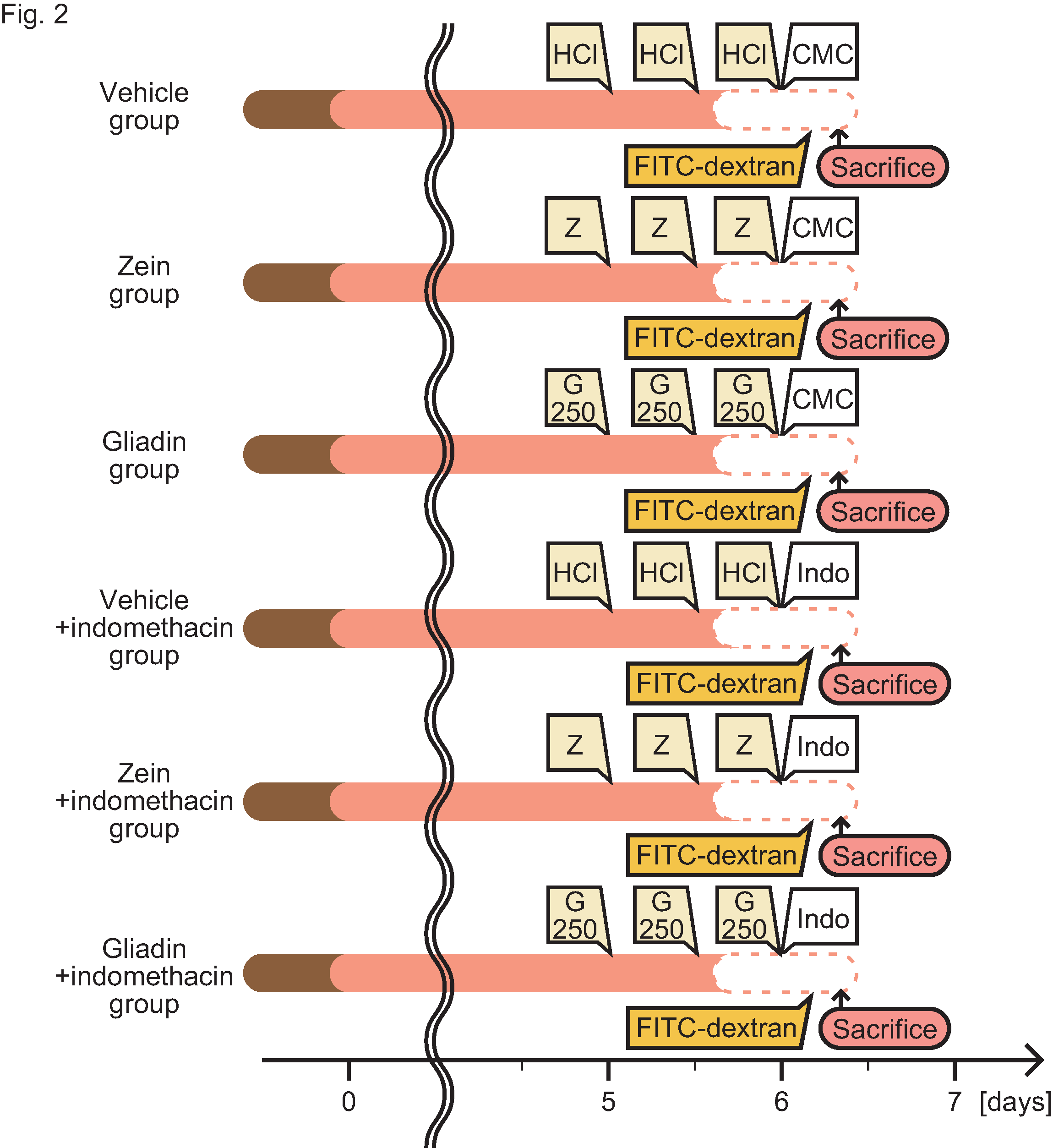

Supplement: S3 Fig — CMC: carboxymethylcellulose, Z: oral administration of zein, G: oral administration of gliadin. (TIF) [file pone.0211436.s004.tif]

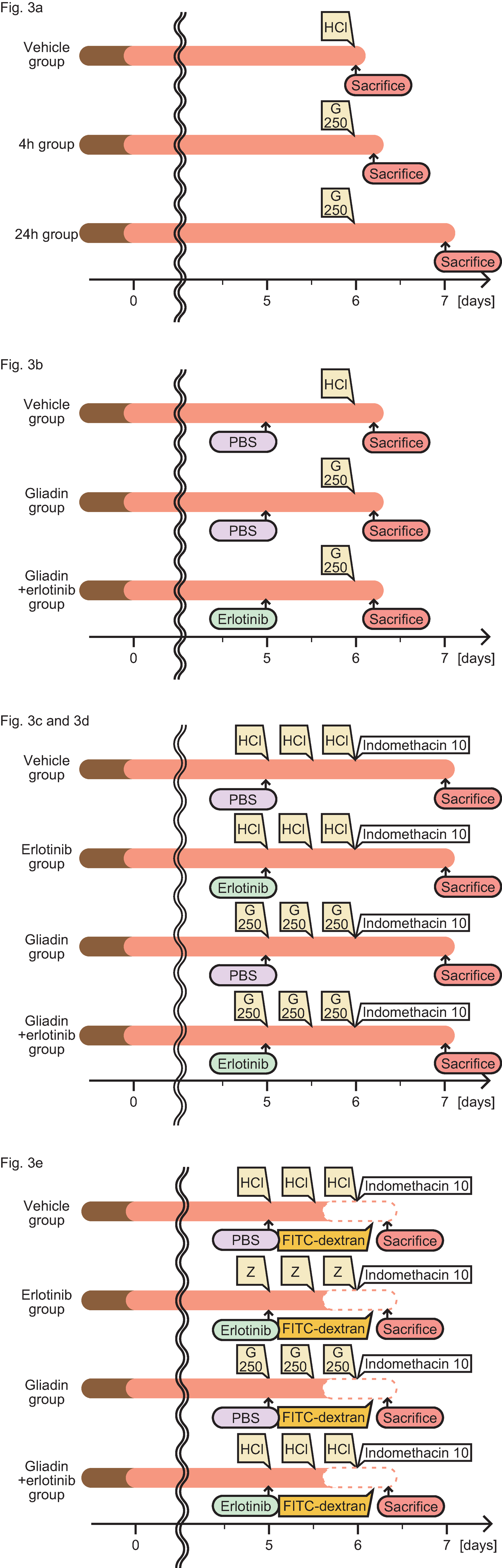

Supplement: S4 Fig — G: oral administration of gliadin, Z: oral administration of zein. (TIF) [file pone.0211436.s005.tif]
